# Annotations

**Published:** 1899-06-10

**Authors:** 


### Annotations.

### The Chigoe.

The spread of the chigoe, or, as it is commonly called, the chiggir, to India is a matter of very considerable importance to the inhabitants of that country. It serves well to illustrate how the free intercommunication between far distant countries, which is so characteristic of this end of the century, brings its own penalty in the way of a wide diffusion of certain forms of disease which have hitherto been credited with being peculiar to more or less limited regions. The *pulex penetrans* is a native of South America and the West Indies, whence it is stated to have been conveyed in the course of trade to the West Coast of Africa. How it crossed Africa is, of course, unknown, but it is not at all difficult to understand that a grub like this, which burrows under the skin of man and then lies dormant for a considerable time during the development of its larvæ, may easily be carried over considerable distances along a trade route. Each individual sufferer, no doubt, only goes backwards and forwards along his own little track, but fresh individuals take up the infection and carry it further, and thus the disease spreads. At any rate, it is now very prevalent among the Indian coolies working on the Uganda Railway, and the Indian Government is finding it necessary to take special precautions to prevent its introduction into India.

### Apoplexy and First Aid.

The tragic death of Dr. Wallace, who was struck down by apoplexy while speaking in the House of Commons, and died within twelve hours of the seizure, may perhaps be taken by some to point the moral that when men arrive at the age of brittle arteries they should retire from the excitements of party life. We could not accept any such limitation to the use to which every man may put his powers. The mere preservation of one's own life by no means appeals to us as a high ambition, and surely an elderly man may quite as fitly run some risk in placing his knowledge and experience at his country's service as a young man may in fighting battles for his country's good. And he should get the credit of it. There is more heroism than some are ready to allow in our elderly politicians. If life is everything, and the preservation of life one's highest aim, it must, indeed, be counted heroic that men over sixty years of age—men often with failing hearts, with imperfect kidneys, and with arteries becoming every year more brittle and inelastic—should expose themselves, for the sake of the principles they have embraced, to the excitement of party life, to the buffetings of party rancour, to the turmoil of elections, and what perhaps is more injurious to nutrition and more effective in shortening life than all the rest, to the disappointments and ingratitude of a political career. It is not every day that these—shall we call them heroes?—drop in their tracks, but medical men who know their secrets know well enough how many elderly men, some of whom may, perchance, be ticketed by the public as cranks and faddists, and may have served for years as figures of fun for the comic papers, walk down day after day to the House of Commons at the call of what they consider duty, literally carrying their lives in their hands, and dependent upon the accidents of debate whether or no they will come out again alive. Under such circumstances, would it not be well that some better arrangements should be made in order that our legislators should receive immediate medical attention on the occurrence of any accident while within the precincts of the House? And, further, would it not be well that the members of the House, or at least its attendants and the police on duty there, should possess some elementary knowledge how to proceed in case of sudden illness? The action of our elected representatives, and the amount of unwisdom displayed in the face of an emergency such as this, would be comic were it not so overwhelmingly pathetic. Think of what happened. An elderly man staggered in his speech, sat down, and rapidly lost all power and consciousness. What was the matter? It might be a sudden failure of the heart, or it might be apoplexy—an artery bursting, perhaps, within the brain, and tearing up its substance. Whatever was the matter, and we do not expect our legislators to be diagnosticians, to keep the patient quiet was the first essential, and when lifted he should have been carried by many hands so as to be disturbed as little as possible. Any policeman who had gone through an ambulance course would have known as much as this, and even the instinct of the sufferer led him to say in his half unconsciousness, "Let me alone." Instead of which, we are told that he was picked up by an active Member, who "took hold of the bulky and heavy form of the fallen man, and carried him out of the House." A triumph of athleticism!—but, of all things in the world, what a thing to do!

### Hospital Milk.

It is a very old, as well as a somewhat silly joke, which describes how an Irishman lengthened his blanket by cutting a piece off from the top, and sewing it on at the bottom. But it is something very analogous to this, and almost as foolish, which is done by certain hospitals, which, while expending much of their energies on the treatment of tuberculous complaints, supply their patients with milk which, for aught they know, may itself be infected with the germs of the very disease which they are endeavouring to cure. We have several times pointed out that the action, or rather the want of action, of many hospitals in regard to protecting their patients from this source of infection casts a shadow of unreality over the whole of the movement which is now so much talked about for the suppression of tuberculosis; for if institutions, such as hospitals, in which more than in any others medical influence ought to be strong, still continue to act as if infection by milk were a myth, a mere phantasy of the imagination, how are we to expect the public at large to take the matter seriously? We are glad to see that the *British Medical Journal* now joins in the protest against the continued indifference of certain hospital boards in regard to this important matter. All milk supplied to hospitals ought to be bought under a warranty of having been procured from healthy cows. If milk infection is a mere myth let someone of influence arise and prick the bubble; but if it is true, in heaven's name let us deal with the problem seriously, and as befits those to whom ordinary people may well look for example.

